# The prevalence and co-existence of geriatric syndromes in older patients with dementia compared to those without dementia

**DOI:** 10.1007/s40520-024-02724-8

**Published:** 2024-03-13

**Authors:** Pinar Soysal, Lee Smith

**Affiliations:** 1 Faculty of Medicine, Department of Geriatric Medicine, Bezmialem Vakif University, 34093 Fatih Istanbul, Turkey; 2 Centre for Health, Performance and Wellbeing, Anglia Ruskin University, Cambridge, UK; 3 Department of Geriatric Medicine, Faculty of Medicine, Bezmialem Vakif University, Adnan Menderes Bulvarı Vatan Street, 34093 Fatih Istanbul, Turkey

**Keywords:** Dementia, Geriatric syndromes, Polypharmacy, Malnutrition, Frailty, Older adults

## Abstract

**Background:**

This study aims to compare frequency and coexistence of geriatric syndromes in older patients with dementia to those without dementia.

**Methods:**

1392 patients admitted to geriatric outpatient clinics were evaluated. Evaluations for eleven geriatric syndromes including polypharmacy, malnutrition, fraility, sarcopenia, dysphagia, urinary incontinence, fear of falling, falls, insomnia, excessive daytime sleepiness, and orthostatic hypotension (OH) were carried out in consultation with the patient and the caregiver. Two groups with and without dementia were matched according to age and gender using the propensity score matching method.

**Results:**

A total of 738 patients, 369 with dementia and 369 without dementia were included, of whom 70.1% were female and the mean age was 80.5 ± 6.8. Polypharmacy, malnutrition, frailty, sarcopenia, dysphagia, fear of falling, and excessive daytime sleepiness were significantly higher in patients with dementia (p < 0.05). There was no difference between OH, urinary incontinence and insomnia between groups (p > 0.05). The co-existence of 0, 1, 2, 3, 4 and ≥ 5 geriatric syndromes in the same patient was 4.3%, 10.2%, 11.8%, 16.8%, 13.4% and 43.7% in non-dementia patients, respectively; 2.4%, 7.2%, 9.6%, 8.3%, 10.4% and 62.1% in those with dementia, respectively (p < 0.05).

**Conclusion:**

The presence and co-existence of geriatric syndromes is common in patients with dementia. These geriatric syndromes should be examined by clinicians and healthcare professionals who work with the demented population, so that more successful management of dementia patients may be achieved.

## Introductıon

Geriatric syndromes are serious conditions with significant effects on functionality and quality of life [1]. Geriatric syndromes, that are multifactorial in nature, are closely related to hospitalization, healthcare utilization, healthcare costs and increased mortality [1, 2]. Estimates suggest that 20% of those aged 60–69 years do not have any geriatric syndrome, while 48% of those aged ≥ 80 years have more than four geriatric syndromes at the same time [2]. Clinicians managing the care of older people, such as geriatricians, primary physicians, cardiologists, oncologists, and neurologists, should be aware of these syndromes.

Loss of functionality due to cognitive impairment in patients with dementia is common, such loss of functionality likely causes complications with basic activities of daily living such as bathing, dressing, and eating, and instrumental activities of daily living such as shopping, taking medications, and coping with finances [3]. Furthermore, several other factors complicate the management of dementia for patients, caregivers and clinicians such as the presence of geriatric Syndromes [4]. Geriatric syndromes such as polypharmacy, malnutrition, fraility, sarcopenia, dysphagia, urinary incontinence, fear of falling, falls, and sleep disorders are common from the early stages of dementia, all of which adversely affect disease progression [5, 6]. Although literature suggests that the frequency of geriatric syndromes is likely high in older dementia patients, there is no study to compare the frequency of geriatric syndromes between those with and without dementia. Findings from comparing geriatric syndromes between these two groups may subsequently be used to raise clinical awareness of such sydromes among those with dementia.

Therefore, the aim of the present study was to compare age- and sex-matched older patients with dementia to those without dementia in terms of the frequency and coexistence of geriatric syndromes.

## Materıals and methods

### Patients

Between January 2019 and October 2021, 1392 patients admitted to geriatric outpatient clinics were evaluated. Patients ≥ 60 years of age were included in the study. Ethical approval was obtained by the ethics committees of Bezmialem Vakif University (E-54022451-050.05.04-20940) and informed consent was provided by each participant or a legal guardian before participating in the study.

### Exclusion criteria

Patients who had severe illness that may impair their general health status, such as acute cerebrovascular event, sepsis, acute renal failure, acute coronary syndrome, and acute respiratory failure; those who declined to participate; those with neuromuscular disease, which causes obstacle to walking and immobile patients; those in delirium at the time of assessments; those with mild cognitive impairment and with severe dementia were excluded. Those with severe vision and hearing impairment that prevent communication and understanding commands during the examination were also excluded.

### Patients’ characteristics

Age, gender, marital status, living status, smoking, caring and driving status of all patients were recorded. The patients’s comorbidities including hypertension, coronary artery disease, diabetes mellitus, congestive heart failure, cerebrovascular events, osteoartiritis, Parkinson’s Disease, Chronic obstructive pulmonary disease (COPD), and Benign Prostatatic Hyperlasia (BPH) were also recorded. Charlson Comorbidity Index was also calculated for all patients.

### Diagnosis of dementia

Either a geriatrician, psychologist or gerontologist interviewed family members or a caregiver of each included patient and through doing so obtained information about the cognitive functioning of the participants and their activities of daily living in recent years. Moreover, through direct assessment a neurocognitive examination on the patients who may have cognitive impairment was carried out. Dementia was diagnosed according to the Diagnostic and Statistical Manual of Mental Disorders—Fifth Edition major cognitive impairment diagnostic criteria. All patients with dementia underwent neuroimaging, including cranial magnetic resonance imaging or computed tomography, to rule out other causes of cognitive impairment (such as intracranial hemorrhage, brain cancer).

**Diagnosis of geriatric syndromes** [2, 5–8]

Evaluations for the following eleven geriatric syndromes were made in consultation with the patient and caregiver:

1. **Polypharmacy:** It is stated as concomitant five or more drug usage.
2. **Malnutrition:** Mini Nutritional Assessment Scale total score < 17.
3. **Frailty:** A modified Fried physical frailty scale was used to evaluate frailty, which was defined according to physical model and the presence of three or more of the following criteria: weight loss, exhaustion, low physical activity, slowness, and weakness. Low physical activity was considered positive in patients who spend the majority of their time sitting or rarely have short walks in the past year.
4. **Sarcopenia:** SARC-F score ≥ 4.
5. **Dysphagia:** Eating Assessment Tool (EAT-10) score ≥ 3.
6. **Orthostatic hypotension (OH):** A decrease in systolic and/or diastolic blood pressure of ≥ 20 mmHg and/or ≥ 10 mmHg, respectively, when one transitions from the supine to an upright position.
7. **Urinary incontinence:** The involuntary leakage in the last 3 months except when urinary tract infection was present.
8. **Falls:** The patient fell in the previous year, other than slipping on the wet floor.
9. **Fear of falling:** The Falls Efficacy Scale—International > 16.
10. **Insomnia**: Insomnia Severity Index score ≥ 8.
11. **Excessive daytime sleepiness:** Epworth Sleepiness Scale score ≥ 11.

## Statistical analyses

Analysis of the data was carried out using SPSS for Windows 22 package program (SPSS Inc., Chicago, IL, USA). There were 1392 patients, 498 of whom had dementia at the beginning, but two groups with and without dementia were matched according to age and gender using the propensity score matching method. Thus, two groups of 369 people who were 100% identical according to age and gender were formed, and all other analyzes were performed on these two groups. Continuous variables were assessed as means and standard deviations and evaluated using the Kolmogorov–Smirnov test for normal distribution. In case of non-normal distribution, continuous variables were evaluated by Mann–Whitney U test. Differences between categorical variables were evaluated by Chi-square and Fisher’s exact Chi-square tests. When more than 25% of the cells were less than 5, the Fisher's exact test was used. A p value < 0.05 was considered significant.

## Results

A total of 738 patients, 369 with dementia and 369 without dementia, of whom 70.1% were female and the sample had a mean age 80.5 ± 6.8 years, were included in the study. Patients’ characteristics are shown in Table 1. There were no significant differences between the two dementia groups in terms of education, or comorbidities including coronary artery disease, diabetes mellitus, congestive heart failure, cerebrovascular disease, peripheral artery disease, COPD, BPH, (p > 0.05). While Parkinson's disease was more common in dementia patients, hypertension and osteoarthritis were more common in non-dementia patients (p < 0.05) (Table 1).

**Table 1:** Demographic and Clinical Characteristics of the participants

Characteristic	Dementia (n=369)	Non-dementia (n=369)	
			p value
Age (mean, SD)	80.53±6.868	80.53±6.868	
Gender, female, %	70.1	70.1	
Marital status, %			
Married	39.6	70.1	0.076
Widowed	46.3	3.6	
Divorced	1.4	0.5	
Single	12.7	0.7	
Living status, %			
Alone	6.8	13.1	**0.002**
With spouse	37.8	44.7	
With children	46.8	37.7	
With caregiver	7.8	4.1	
With grandchildren	0.3	0	
Other	0.5	0.3	
Smoking status, %			
No	71.7	67.1	0.084
Past smoking	21.3	26.5	
Still	6	6.4	
Pasive	1.1	0	
Driving, %			
Never driven	86,6	85.9	**0.038**
Past driving	11.2	8.6	
Still driving	2.2	5.5	
Comorbidities, %			0.008
Hypertension	64.9	74.1	**0.004**
Diabetes mellitus	36.9	37.8	0.428
Congestive heart failure	12.5	10.1	0.179
Cerebrovascular events	13	11.1	0.249
Coronery artery disease	21.7	20.1	0.325
COPD	6.5	8.4	0.206
Peripheral artery disease	3.5	3.2	0.497
Osteoartiritis	9.7	14.9	**0.021**
Parkinson’s disease	12.2	7.9	**0.033**
BPH	7	8.6	0.247
Charlson comorbidity index	2.6±1.3	2.9±1.4	0.186
Education (years)	5.2±2.9	5.0±2.8	0.898

Bold p values show statistically significant results

*BPH* benign Prostatatic Hyperlasia; *COPD* chronic obstructive pulmonary disease

**Fig.1:**
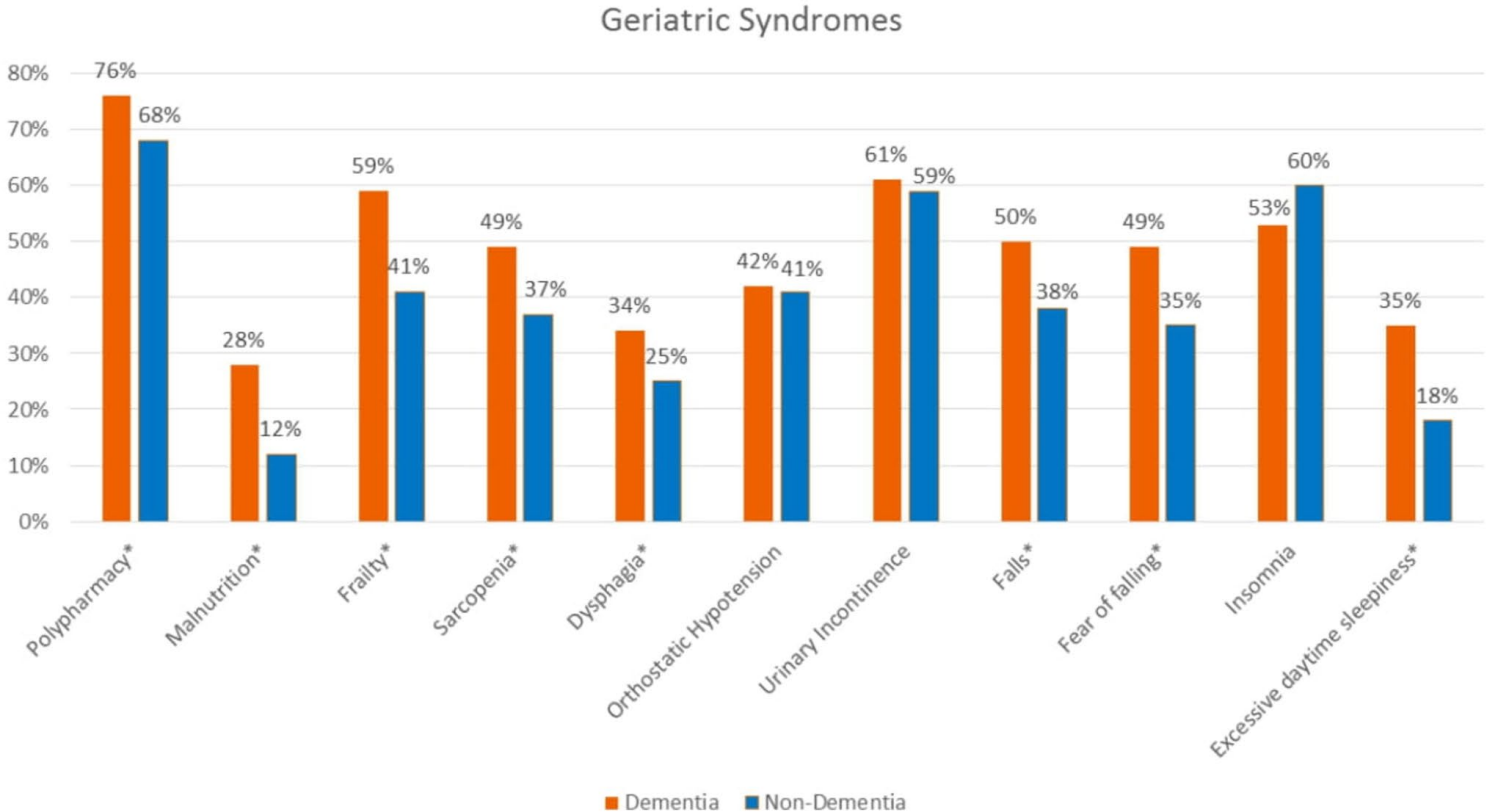
Frequency of geriatric syndromes according to the presence of dementia

Polypharmacy, malnutrition, frailty, sarcopenia, dysphagia, fear of falling, and excessive daytime sleepiness were significantly higher in patients with dementia (*p* < 0.05). There was no difference between OH, urinary incontinence and insomnia between those with and without dementia (*p* > 0.05). The most common geriatric syndromes with a frequency of over 50% in dementia patients were polypharmacy, frailty, falls, urinary incontinence, and insomnia. In patients without dementia, the most common geriatric syndromes, over 50%, were polypharmacy, urinary incontinence and insomnia (Fig. 1).

**Fig.2:**
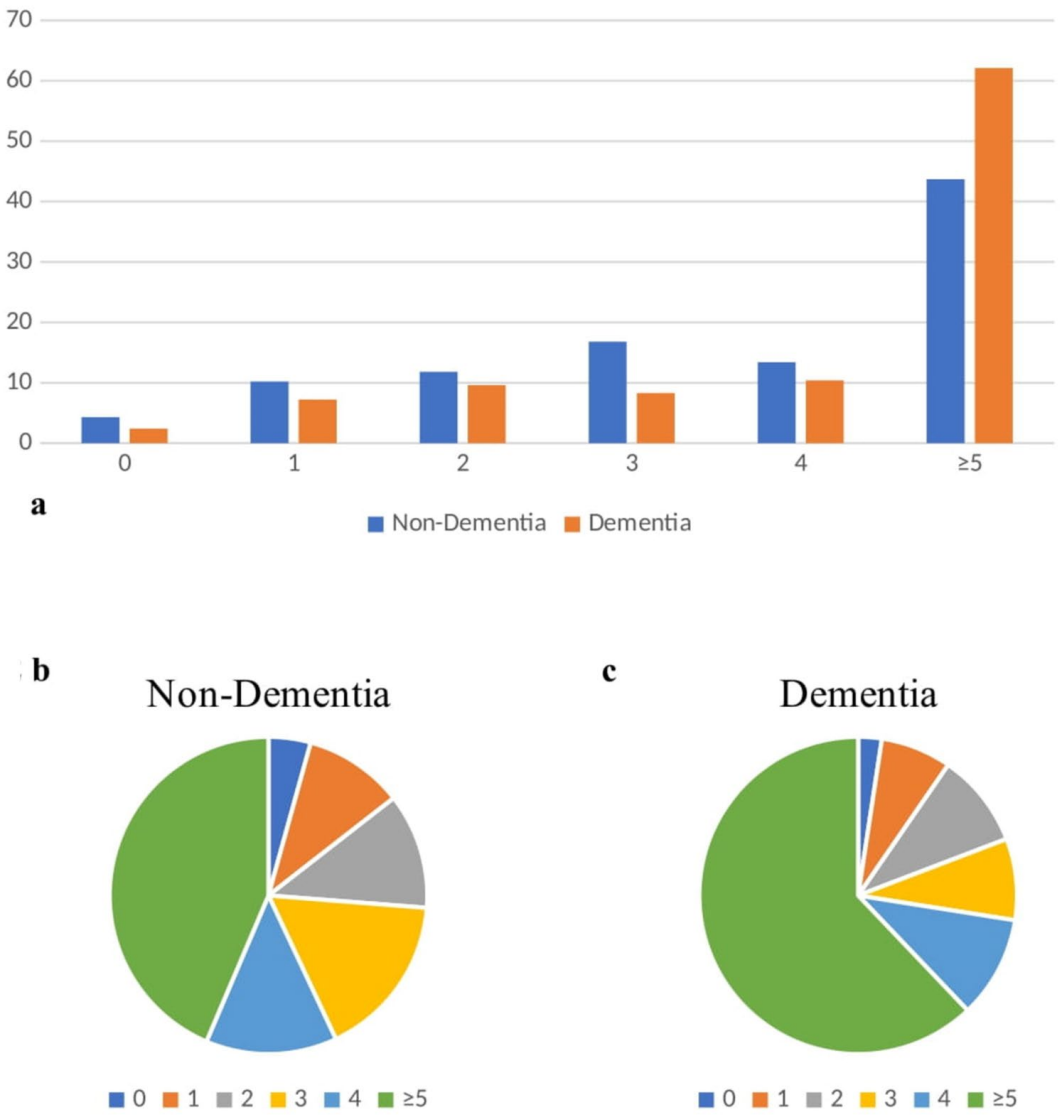
The number of geriatric syndromes according to the presence of dementia

The coexistence of 0, 1, 2, 3, 4 and ≥ 5 geriatric syndromes in the same patient was 4.3%, 10.2%, 11.8%, 16.8%, 13.4% and 43.7% in non-dementia patients, respectively; those with dementia were 2.4%, 7.2%, 9.6%, 8.3%, 10.4% and 62.1%, respectively (Fig. 2a–c).

## Discussion

The frequency of geriatric syndromes and their associations is generally high in dementia patients. Polypharmacy, malnutrition, frailty, sarcopenia, dysphagia, fear of falling, and excessive daytime sleepiness are more common in those with dementia than in those without; OH, urinary incontinence and insomnia are as common in the older patients without dementia as those with dementia. One out of every two non-dementia patients and almost three out of every four dementia patients have the coexistence of ≥ 4 geriatric syndromes.

Each geriatric syndrome impairs the quality of life and complicates the care and management of older patients [2]. Therefore, multiple syndromes in the same patient means that his/her care becomes more complicated to manage for both the clinician and the caregiver. It is also important that the geriatric syndromes that geriatricians manage are known by other physicians who manage diseases that increase with age. In this study, polypharmacy, malnutrition, frailty, sarcopenia, dysphagia, fear of falling, and excessive daytime sleepiness were much higher in dementia patients. Moreover, the simultaneous presence of 5 geriatric syndromes in over 60% of dementia patients is an indication of how complex the management of older patients with dementia may be.

Polypharmacy was the most frequently detected geriatric syndrome in our study (76% in patients with dementia and 68% in patients without dementia). Polypharmacy is associated with hospitalization, admission to emergency departments and increased mortality in older adults with or without dementia [9, 10]. In addition, polypharmacy can delay the initiation of dementia treatment; there may also be increased functionality and cognitive decline in those with polypharmacy [9]. Multiple comorbidities and the high frequency of other geriatric syndromes as determined in our study, as well as drugs prescribed for the treatment of them, dementia and neuropsychiatric symptoms, may cause polypharmacy [11]. Therefore, medication should be prescribed on a profit-loss basis; otherwise, inappropriate drug use and polipharmacy itself may increase the risk of development or the severity of other geriatric syndromes such as falls and malnutrition [11].

Dysphagia and malnutrition are more common in dementia patients [12]. The relationship between dementia and malnutrition is complex and people with dementia might be more prone to losing weight for several reasons, including difficulties in activities of daily living, such as shopping and cooking, which may limit food consumption [13]. Further, malnutrition may be affected by behavioral and psychological symptoms of dementia by both reducing food intake and physical activity [14]. Patients may have reduced food intake due to progression of cholinergic deficits and dementia leading to dysphagia and alteration of taste and smell which, in turn, reduces patients’ interest in food [15]. Another important point to note is that acetylcholinesterase inhibitors are associated with weight loss [16]. Finally, malnutrition and dysphagia, common in people with dementia, are associated with more frequent occurrences of neuropsychiatric symptoms and faster cognitive decline, as well as associated with hospitalization and mortality, similar to the general population [17].

In our study, it was found that one in every two dementia patients had a history of falling and fear of falling. The relationship between cognitive impairment and falls/fear of falling has been widely studied in recent years. According to the studies, falls/fear of falling and dementia have similar risk factors such as physical inactivity and decreased socialization [18]. Fear of falling and falls are strongly related to inadequacy in some cognitive function domains, such as executive function and processing [19]. In addition, extrapyramidal signs such as parkinsonism, rigidity, and postural instability, which are not only common in patients with Lewy body dementia, but also in patients with vascular and Alzheimer's type dementia, may cause falls and fear of falling more frequently in dementia [20, 21]. Moreover, factors that may negatively affect attention, such as visuospatial changes and neuropsychiatric symptoms, use of psychotropic drugs, or sleep disorders may contribute to the increase in fear of falling in dementia patients [22].

Falls, fear of falling, malnutrition, dysphagia and even polypharmacy are important components of the development of sarcopenia [23]. There are only a few studies examining why sarcopenia is more common in patients with dementia. However, in a few studies, loss of appendicular muscle mass was found to be associated with AD-related brain atrophy, and total skeletal muscle mass including fat free mass was reduced in patients with dementia compared to those with mild cognitive impairment [24].Thus, decreased brain neuronal volume in those with dementia may result in a reduction in muscle mass [24]. All the above-mentioned geriatric syndromes, especially sarcopenia, are also strongly associated with frailty [25]. Slow walking and involuntary weight loss that begin years before the diagnosis of dementia, are important components of frailty [26].This shows that frailty may trigger the underlying mechanisms bringing about the onset of dementia, which increases the vulnerability of the individual to the disease [25]. Although the mechanisms underlying these associations are not yet clear, oxidative stress and inflammation are suggested to play a role [25].

The finding that urinary incontinence, OH, and insomnia are as common in people without dementia as those with dementia indicates that non-neurogenic causes in the elderly are also effective in the development of these three factors. Another explanation of this finding may be that we included only early and middle stage of dementia patients and excluded severe dementia. Although sleep problems are observed from the early stages of neurodegenerative diseases, no difference in dementia status was observed in relation to insomnia, while excessive daytime sleepiness was found more frequently in dementia. In a study evaluating the impact of insomnia and excessive daytime sleepiness in the elderly, excessive daytime sleepiness, but not insomnia, was observed to be an important risk factor for cognitive decline and the onset of dementia [27]. Importantly, excessive daytime sleepiness is frequently seen in Parkinson's disease, another neurodegenerative disorder [28]. In our study, excessive daytime sleepiness may have been observed frequently, since Parkinson's disease was more common in patients with dementia.

Findings from the present study must be observed in light of its limitations. First, the study was cross-sectional and not analyzed according to dementia subtypes. Next, the present study did not include MCI or advanced dementia and did not neuropathologically confirm dementia diagnosis. Another limitation maybe that we did not evaluate the drugs types, such as, antipsychotic or antidepressant. Last, delirium and geriatric depression, two important geriatric syndromes, were not evaluated in the patients. Strengths of the present study include the adequate sample size, the exact matched age and gender of those with and without dementia, and the simultaneous evaluation of multiple geriatric syndromes. However, delirium could not be included in the present study because this stuy included an outpatient sample, only.

## Conclusion

The presence and coexistence of geriatric syndromes is common in patients with early and middle stages of dementia. These geriatric syndromes, which may be triggered by the onset of cognitive impairment and the dependency on activities of daily living, have a common point in their etiopathogenesis and are closely related to each other, increase mortality and morbidity in patients and complicate patient management. Therefore, these geriatric syndromes, which are the preoccupation of geriatricians, should at least be questioned by other clinicians and cooperated with geriatricians, so that more successful management of dementia patients may be achieved.

## Data Availability

No datasets were generated or analysed during the current study
